# Evaluation of 14 Organic Solvents and Carriers for Screening Applications in Zebrafish Embryos and Larvae

**DOI:** 10.1371/journal.pone.0043850

**Published:** 2012-10-17

**Authors:** Jan Maes, Lien Verlooy, Olivia E. Buenafe, Peter A. M. de Witte, Camila V. Esguerra, Alexander D. Crawford

**Affiliations:** Laboratory for Molecular Biodiscovery, Department of Pharmaceutical and Pharmacological Sciences, University of Leuven, Leuven, Belgium; Alexander Flemming Biomedical Sciences Research Center, Greece

## Abstract

Zebrafish are rapidly growing in popularity as an *in vivo* model system for chemical genetics, drug discovery, and toxicology, and more recently also for natural product discovery. Experiments involving the pharmacological evaluation of small molecules or natural product extracts in zebrafish bioassays require the effective delivery of these compounds to embryos and larvae. While most samples to be screened are first solubilized in dimethyl sulfoxide (DMSO), which is then diluted in the embryo medium, often this method is not sufficient to prevent the immediate or eventual precipitation of the sample. Certain compounds and extracts are also not highly soluble in DMSO. In such instances the use of carriers and/or other solvents might offer an alternative means to achieve the required sample concentration. Towards this end, we determined the maximum tolerated concentration (MTC) of several commonly used solvents and carriers in zebrafish embryos and larvae at various developmental stages. Solvents evaluated for this study included acetone, acetonitrile, butanone, dimethyl formamide, DMSO, ethanol, glycerol, isopropanol, methanol, polyethylene glycol (PEG-400), propylene glycol, and solketal, and carriers included albumin (BSA) and cyclodextrin (2-hydroxypropyl-beta-cyclodextrin, or HPBCD). This study resulted in the identification of polyethylene glycol (PEG400), propylene glycol, and methanol as solvents that were relatively well-tolerated over a range of developmental stages. In addition, our results showed that acetone was well-tolerated by embryos but not by larvae, and 1% cyclodextrin (HPBCD) was well-tolerated by both embryos and larvae, indicating the utility of this carrier for compound screening in zebrafish. However, given the relatively small differences (2–3 fold) between concentrations that are apparently safe and those that are clearly toxic, further studies – e.g. omics analyses –should be carried out to determine which cellular processes and signalling pathways are affected by any solvents and carriers that are used for small-molecule screens in zebrafish.

## Introduction

Following two decades of rapidly increasing popularity as a model organism for developmental and biomedical genetics, zebrafish (*Danio rerio*) have recently also emerged as an ideal system for *in vivo* studies using small molecules. In the area of chemical genetics, zebrafish are now well-validated as an animal model for the utilization of small molecules to eludicate biological function in a manner similar to traditional genetic analysis, as well as for deciphering the mechanism of action of bioactive compounds [1], [2], [3]. For drug discovery applications, zebrafish assays and disease models have been established in a number of indication areas to search for bioactive compounds with therapeutic applications [4], [5]. More recently, zebrafish-based assays have proven useful for structure-activity relationship studies [6]. In the area of toxicology, a number of *in vivo* assays based on zebrafish embryos and larvae are now available to examine the potential toxicity of compounds early in the drug discovery process [7], [8], as well as to assess the biological impact of chemicals in the environment [9]. Zebrafish are now also finding utility for natural product discovery, where they can be used for the bioassay-guided isolation of drug- like compounds from secondary metabolite extracts obtained from plants or other organisms [10], [11], [12].

For all of these applications, compounds and/or natural product extracts need to be delivered to zebrafish embryos and larvae to exert their bioactivity. One possible method for this purpose is microinjection, which is widely used to deliver antisense oligonucleotides [13], *in vitro*-transcribed RNA [14] and DNA transgene vectors [15] to zebrafish embryos during early cleavage stages. Microinjection can also be used to deliver compounds to the circulation of embryos and larvae, as was recently carried out for a series of poorly absorbing, QT-prolonging drugs to examine their ability to induce bradycardia in zebrafish [16]. Nevertheless, most small molecules are relatively cell- permeable and do not require this effective but lower-throughput mode of application. Also, only very small volumes can be delivered by microinjection (1–2 nanoliters on average), meaning that only limited amounts of compound are introduced, which may be diluted below their effective concentration range by the developmental stage in which their activity is to be assessed.

For most large-scale compound screens, therefore, small molecules are delivered to zebrafish embryos and larvae by aqueous exposure. As many compounds exhibit limited solubility in aqueous solution, the use of organic solvents is required to create stock solutions that can then be diluted in the embryo medium. The concentration of compounds in these stock solutions can range up to 10 mM or 10 mg/ml for most small molecules, depending on the dilution factor and the final concentration to be tested. To ensure detection of minor bioactive constituents, crude natural product extracts are often screened in zebrafish bioassays [10], [11], [12] at final concentrations of up to 200 µg/ml, requiring a stock solution of 20 mg/ml that is then diluted 1∶100 in the embryo medium. For the analysis of seizure-like behavior and locomotor activity in larvae at 7 days post-fertilization (dpf), both proconvulsants and anticonvulsants must sometimes be used at final concentrations in the low millimolar range to achieve the desired effect [17], [18], [19].

Although DMSO is widely used as a solvent for compound screening, both in zebrafish and in cell-based assays, this solvent does not dissolve all compounds. Often, other solvents must be used to achieve sufficient solubility, and in some cases, carriers may be required to maintain a certain level of compound in solution upon final dilution in aqueous media. Although initial toxicological data have been obtained in zebrafish for DMSO and ethanol in particular, most other solvents and carriers have not yet been tested in zebrafish. To examine the suitability of these substances for zebrafish-based compound screening experiments, we carried out a comparative toxicity study in zebrafish embryos and larvae of the solvents acetone, acetonitrile, butanone, dimethyl formamide, DMSO, ethanol, glycerol, isopropanol, methanol, polyethylene glycol (PEG-400), propylene glycol, and solketal, and the carriers albumin (BSA) and cyclodextrin (2-hydroxypropyl-beta-cyclodextrin, or HPBCD).

## Materials and Methods

Zebrafish embryos of the AB wild-type strain (originally obtained from the Zebrafish International Resource Center, Eugene, Oregon, USA) were raised at 28°C. Zebrafish husbandry, embryo collection, and embryo and larva maintenance were performed as described [20], [21]. Toxicity assays were standardly performed in 24-well microtiter plates (wrapped with Parafilm to limit solvent evaporation) using 10 embryos per well in 1 ml of 0.3× Danieau's medium (17 mM NaCl, 2 mM KCl, 0.12 mM MgSO4, 1.8 mM Ca(NO3)2 and 1.5 mM HEPES, pH 7.6). Each experiment was repeated 3 times for a total of 30 embryos or larvae analyzed per solvent per developmental staged tested. Data were only recorded for experiments in which the percentage of normal embryos or larvae in the control group was at least 90%. Embryos and larvae were exposed to solvents and carriers at 2–4 cells, 4 hpf, and at 1, 2, 3, 4, and 7 dpf and evaluated for signs of toxicity 24 hours later. In determining the maximum tolerated concentration (MTC) for each solvent and carrier, all post-exposure embryos and larvae were allowed to develop in larva medium to 9 dpf, so as to detect any deleterious effects appearing after this 24-hour window. Solvents and carriers were obtained from the following suppliers: acetone (Chemlab, Zedelgem, Belgium), acetonitrile (Acros Organics, Geel, Belgium), albumin (BSA, Sigma-Aldrich, Bornem, Belgium), butanone (Riedel-de Haën, Seelze, Germany), cyclodextrin (2- hydroxypropyl-beta-cyclodextrin, Sigma-Aldrich), dimethyl formamide (Acros), DMSO (Agros), ethanol (Fisher Scientific, Doornik, Belgium), glycerol (Acros), isopropanol (Chemlab), methanol (Chemlab), polyethylene glycol-400 (Fluka, Bornem, Belgium), propylene glycol (Certa, Eigenbrakel, Belgium), solketal (Merck, Overijse, Belgium). Statistical analyses were done using chi-square in Microsoft Excel.

## Results and Discussion

Twelve solvents and 2 carriers – chosen based solely on their potential utility for the solubilization and delivery of a structurally diverse range of small molecules – were analyzed for their effects on zebrafish embryos and larvae. The solvents included acetone, acetonitrile, butanone, dimethyl formamide, DMSO, ethanol, glycerol, isopropanol, methanol, polyethylene glycol (PEG-400), propylene glycol, and solketal, and the carriers included albumin (BSA) and cyclodextrin (2-hydroxypropyl-beta-cyclodextrin, or HPBCD). As a solvent or carrier concentration of 1–2% is practical for small-molecule screens in zebrafish, and as pilot experiments indicated the majority of these solvents and carriers to be toxic above 2%, a range from 0.5% to 2.5% was chosen for their final concentrations in embryo medium. For this initial study, the effects of a 24-hour exposure to each solvent and carrier was analyzed using embryos and larvae at the following developmental stages: 2–4 cell, 4 hours-post fertilization (hpf), and 1, 2, 3, 4, and 7 days post-fertilization (dpf). No post-exposure effects until 9 dpf were detected at the MTCs shown (Table 1). Phenotypic effects were recorded at the lowest concentration for each solvent, and at each developmental timepoint, in those cases where at least 50% of embryos or larvae were affected (Table 2).

**Table 1:** Maximum-tolerated concentrations of solvents and carriers in zebrafish embryos and larvae.

Solvent/carrier	2–4 cell	4 hpf	1 dpf	2 dpf	3 dpf	4 dpf	5 dpf	7 dpf
Acetone	2	2	1.5	1.5	≥2.5	1.5	<0.5	<0.5
Acetonitrile	<0.5	0.5	1	0.5	1	0.5	0.5	0.5
Albumin (BSA)	<0.5	<0.5	<0.5	0.5	0.5	1.5	≥2.5	≥2.5
Butanone (MEK)	0.5	1	1	1.5	1.5	1.5	<0.5	0.5
Cyclodextrin (HPBCD)	1	0.5	1	1.5	1.5	1	1.5	1
Dimethyl formamide (DMF)	0.5	<0.5	<0.5	0.5	0.5	0.5	0.5	<0.5
Dimethyl sulfoxide (DMSO)	>2.5	>2.5	2	2	1.5	2	2	1.5
Ethanol	1	0.5	1	1.5	1	1.5	0.5	1
Glycerol	1.5	1.5	1.5	1.5	1.5	2	>2.5	>2.5
Isopropanol	0.5	1	0.5	1	1.5	0.5	<0.5	0.5
Methanol	1.5	1.5	1.5	>2.5	2	1	>2.5	2
Polyethylene glycol (PEG-400)	>2.5	>2.5	1.5	>2.5	>2.5	>2.5	>2.5	>2.5
Propylene glycol	>2.5	>2.5	>2.5	>2.5	2	1.5	>2.5	1.5
Solketal	<0.5	0.5	0.5	0.5	0.5	<0.5	<0.5	<0.5

This table depicts the highest concentrations for each solvent and carrier, at each developmental stage tested, at which no phenotypic abnormalities could be observed. Embryos and larvae were incubated in water containing each solvent or carrier at the concentration indicated, for 24 hours beginning at the developmental stage indicated. In determining the maximum tolerated concentration (MTC) for each solvent and carrier, all embryos and larvae were allowed to develop to 9 dpf, so as to detect any deleterious effects appearing after this 24-hour exposure window; none were detected at the MTCs shown.

**Table 2:** Phenotypic effects of solvents and carriers above maximum tolerated concentrations after 24 hours.*

	2–4 cell	4 hpf	1 dpf	2 dpf	3 dpf	4 dpf	5 dpf	7 dpf
Acetone	-	-	-	-	-	-	-	IC (0.5)
Acetonitrile	-	AP IC IP NO SO (1.5)	DD PE (2.5)	PE (1.5)	PE SB (1.5)	IC PE (1.5)	IC PE (1)	IC PE (0.5)
BSA	DD (0.5)	DD (0.5)	-	-	SB (1.5)	IC PE (1.5)	-	-
MEK	-	-	IC MC PE (2)	DD HJ (2.5)	HJ SB (2)	-	-	-
HPBCD	-	-	-	-	-	-	-	-
DMF	EP (1)	AP BC BS CF PE (1)	AP BH IC IM NB PE (0.5)	AP HJ PE (1)	HJ IC PE (1)	AP LP (1)	IM LP PE SB (1.5)	HO LP (0.5)
DMSO	-	-	-	-	NB SB (2.5)	-	GG IC IM LP (2.5)	NB LP (2)
Ethanol	DD (1.5)	AP BS CY IC SO (1.5)	PE (2)	HJ (2)	AH BH EH IC YE (2)	BC HY LP SB (2)	BC LP NB (2.5)	LP (1.5)
Glycerol	AP CB SO (2.5)	AP BS IP (2)	IC IM (2)	IC IM (2)	HO IC IM (2)	-	-	-
Isopropanol	-	IC (1.5)	AP IC (1)	IC LP (2)	BC IC IM HJ PE (2)	-	-	IC LP NB PE (1)
Methanol	IC (2)	IC (2)	-	-	SB (2.5)	-	-	NB LP (2.5)
PEG-400	-	-	-	-	-	-	-	-
Propylene glycol	-	-	-	-	-	-	-	-
Solketal	-	DD (1)	AP BH IC IM NB PE (1)	AP BH IC PE (1)	AH IC IM HJ PE (1)	BC LP (0.5)	NB LP SB+ (0.5)	NB LP (0.5)

* Phenotypes with an average penetrance of at least 50% (in comparison to dead or apparently normal embryos or larvae) are indicated by the following abbreviations: AH, atrial hemorrhage; AP, anterior-posterior axis truncation; BC, body curvature; BH, brain hemorrhage; BS, brain segmentation defects; CB, *cardia bifida*; CF, widened choroid fissure; CY, cyclopia; DD, developmental delay; DH, delayed hatching; EH, elongated heart; EP, epiboly defects; GG, bright green gall bladders; HJ, hypomorphic jaw; HO, hypoactivity; HY, hyperactivity; IC, impaired circulation; IM, impaired motility (lack of touch response); IP, incomplete pigmentation; LP, loss of posture; MC, microcephaly; NB, necrosis in body cavity; NH, necrosis in head; NO, bumpy notochord; PE, pericardial edema; SB, incompletely inflated or uninflated swim bladder; SB+, enlarged swim bladder; SO, somite defects; YE, yolk sac edema. Phenotypes were not recorded for penetrance levels below 50% (i.e. where the majority of embryos or larvae were dead or apparently normal); these cases are denoted by “-”. The lowest concentration (in % of solvent or carrier) at which an average penetrance of at least 50% was observed for the shown phenotypic effects is given in parentheses.

### Acetone

Acetone is a widely used solvent in chemistry and is also used as an excipient in various pharmaceutical formulations. Although not as popular as DMSO in solubilizing compounds for bioassays, acetone has recently been used as a solvent in zebrafish – namely, to solubilize the ectoparasiticide and plasticizer di-n-butyl phthalate (DBP) in an embryotoxicity toxicity assay [22]. In this case, acetone was applied at a concentration of only 0.01%, and negative controls with this level of acetone showed no signs of toxicity.

**Figure 1:**
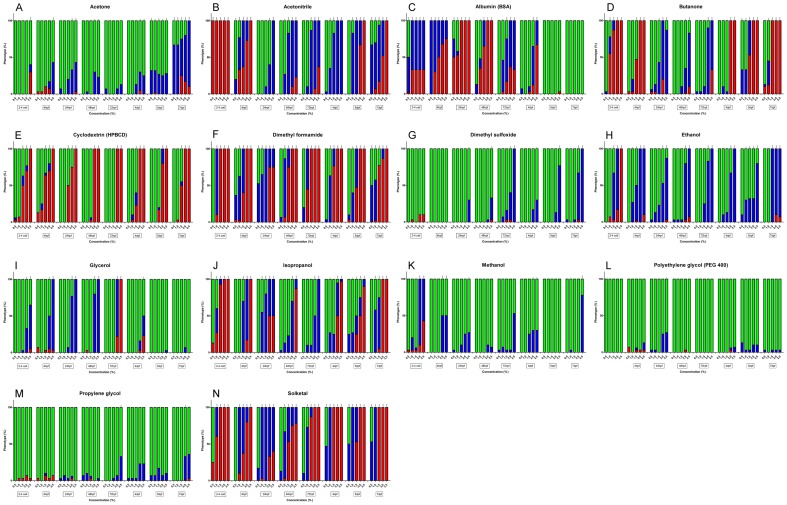
Concentration-dependent toxicities of solvents and carriers in zebrafish embryos and larvae. Effects of various solvent or carrier concentrations on zebrafish embryos and larvae at different developmental stages. Green bars indicate a normal phenotype, blue bars indicate an abnormal phenotype, and red bars indicate embryos or larvae that were dead at the timepoint of analysis. All dysmorphologies and other aberrant phenotypes (e.g. slow heart rate, lack of circulation) are classified as “abnormal” phenotypes in this summary. Each bar represents collated data of 3 separate experiments, for a total of 30 embryos or larvae. The depicted developmental stage (e.g. 4 hpf or 3 dpf) represents the timepoint at which the solvent was added to the medium. Phenotypes were analyzed 24 hours later. Values that are significantly different from the control group are indicated by asterisks: *, p<0.05; **, p<0.01; ***, p<0.001.

In our study, the incubation of zebrafish embryos and larvae with a range of acetone concentrations revealed that early embryos are more tolerant to this solvent than are older larvae (Fig. 1, Table 1). Cleavage-stage embryos developed normally in up to 2% acetone, whereas 7-dpf larvae were strongly affected by concentrations as low as 0.5%. Three-dpf larvae exhibited no visible defects at the highest concentration of 2.5%.

One previous report has investigated the embryotoxicity of acetone [23]. Interestingly, acetone has been found to exert anticonvulsant effects in rodent seizure models, and elevated brain levels of this molecule are thought to be the mechanism of action of the low-carbohydrate, high-fat ketogenic diet used in children to manage drug-resistant epilepsy [24]. In this context, acetone may not be suitable to solubilize compounds being tested in larval zebrafish locomotor assays [17] for their anticonvulsant potential.

### Acetonitrile

The simplest of organic nitriles, acetonitrile is a medium-polarity solvent that can be used to dissolve a wide range of ionic and non-polar compounds, in conjunction with chromatographic analyses as an eluent. Acetonitrile and several other aliphatic nitriles have previously been investigated for their developmental toxicities [25]. The use of acetonitrile in zebrafish assays has not been reported to date.

**Figure 2:**
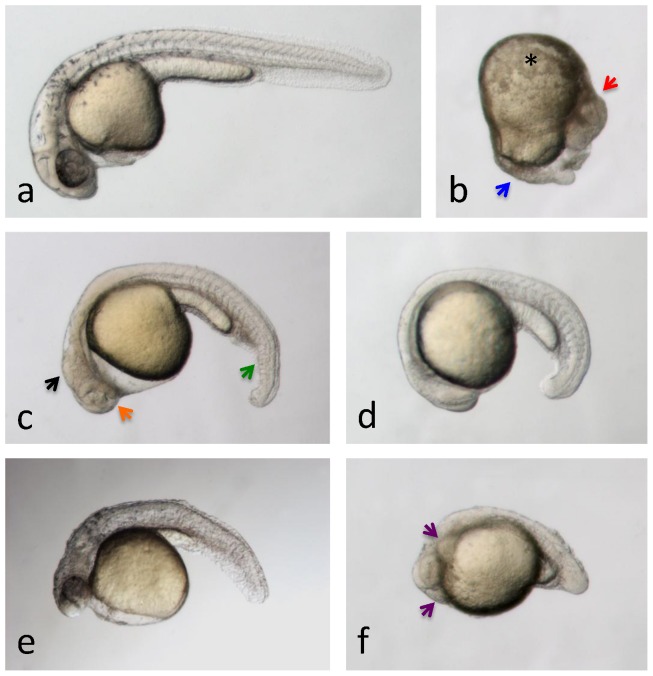
Teratogenic effects of solvents within the first 24 hours post-fertilization. Typical results are shown. Embryos at 1 dpf treated for the previous 24 hours with **a**, embryo medium (control) or **b,** 1% DMF (from 4-cell stage), **c**, 1% DMF (from 4 hpf), **d**, 0.5% albumin (from 4-cell stage), **e**, 1.5% acetonitrile (from 4 hpf), and **f**, 2.5% glycerol (from 4-cell stage). **b**, embryos treated from 4-cell stage with 1% DMF display severe epiboly defects. The eyes (red arrow) and some somites are still visible (blue arrow), but most other structures are no longer clearly discernable. Massive cell death is also present surrounding the yolk (black asterisk). **c**, 1% DMF applied from 4 hpf onwards no longer inhibits epiboly. However, embryos display body curvature and shortening, loss of ventral tail fin (green arrow), no clear brain segmentation (black arrow), pericardial edema and a widened choroid fissure (orange arrow). **d**, embryos treated with 0.5% albumin are morphologically normal but display a significant delay in development (up to 9 hours) relative to control. **e**, 1.5% acetonitrile-treated embryos display anterior-posterior axis truncation, body curvature, microcephaly, lack of a midbrain-hindbrain boundary, incomplete pigmentation in the eyes, trunk and tail, ill-defined somitic boundaries, a bumpy notochord and ‘roughened’ epidermis and fin structures. **f**, embryos treated with 2.5% glycerol display anterior-posterior axis truncation with no extended tail, u-shaped somites, and *cardia bifida* (purple arrows). With the exception of embryo in panel b, which is oriented with anterior to the top, dorsal to the right, all other embryos are depicted with anterior to the left, dorsal to the top.

Acetonitrile is among the less well-tolerated solvents tested in our study. The lowest concentration examined, 0.5%, was completely lethal for cleavage-stage embryos after 24 hours of exposure, and caused unacceptably high levels of abnormalities in 7-dpf larvae after 24 hours of exposure (Fig. 1, Table 1). Larvae at 2, 4 and 5 dpf appeared to tolerate this concentration well, but higher acetonitrile levels (1% and above) were toxic to these animals. Intermediate developmental stages (e.g. 1 and 3 dpf) were found to tolerate up to 1% acetonitrile. The most common defect in embryos and larvae surviving acetonitrile concentrations higher than their respective MTC were impaired circulation and pericardial edema, with swim bladder defects appearing in larvae treated at 3 dpf (Table 2). Embryos treated from 4 hpf onwards with 1.5% acetonitrile displayed anterior-posterior axis truncation, body curvature, microcephaly, lack of a midbrain-hindbrain boundary, incomplete pigmentation in the eyes, trunk and tail, ill-defined somitic boundaries, a bumpy notochord and ‘roughened’ epidermis and fin structures (Fig. 2e, Table 2).

In a whole-embryo culture system, acetonitrile was found to be embryotoxic for rat embryos at a minimum concentration of 40 mM, the highest concentration of the eight aliphatic nitriles tested in the assay [25]. Oral doses given to pregnant rats at gestational day 10 resulted in defects characteristic to embryos exposed to sodium cyanide *in utero* or in culture, suggesting that the maternal conversion of acetonitrile to cyanide may contribute to its developmental toxicity. Whether or not this mechanism plays a similar role for the embryotoxicity of acetonitrile in zebrafish remains to be determined.

### Albumin

Serum albumin, representing over 60% of total plasma protein in humans, has evolved as an endogenous carrier molecule and is responsible for the distribution of a multitude of endogenous compounds with limited water solubility, including hormones, peptides, free fatty acids, calcium and other ions, and bile acids. It is estimated that serum albumin acts as a carrier for over 70% of human drugs. As bovine serum albumin (BSA) is widely used as a carrier for *in vitro* experiments using cultured mammalian cells, we sought to determine its suitability for zebrafish assays.

Our evaluation of BSA in zebrafish revealed an interesting yet puzzling trend – whereas embryos are strongly affected by even the lowest concentrations tested (0.5%), resulting in a strong developmental delay (Fig. 2d, Table 2), larvae at 5 and 7 dpf appeared unaffected by even the highest concentrations (2.5%). Intermediate stages revealed MTCs of 0.5 to 1.5% (Fig. 1, Table 1).

Although such effects of albumin on developing zebrafish have previously not been reported, BSA has been found to enable the delayed *in vitro* fertilization of zebrafish eggs, maintained in Hank's saline, by preventing their activation [26]. One possibility might be that albumin molecules are adsorbing substances in the immediate vicinity of the embryos that are essential for their normal development.

### Butanone

Butanone, also known as methyl ethyl ketone (MEK) or 2-butanone, is a polar solvent with applications in both manufacturing and research. Its use as a solvent for zebrafish assays has not been reported to date.

We found butanone to be moderately well tolerated by embryos and larvae, up to a concentration of 1 or 1.5% for most developmental stages (Fig. 1, Table 1). Interestingly, both cleavage-stage embryos and larvae at 5 and 7 dpf were more sensitive to butanone, with MTCs of 0.5% or lower. Noteworthy phenotypes include microcephaly at 1 dpf, and jaw defects appearing after treatment at 2 and 3 dpf (Table 2).

In both rats and mice, butanone has been found to exhibit embryotoxicity when administered to pregnant females, inducing dysmorphologies such as cleft palate, fused ribs, missing vertebrae, and syndactyly [27], [28]. To date, there have been no other embryotoxicity studies in zebrafish.

### Cyclodextrin

Cyclodextrins are cyclic oligosaccharides widely used as excipients for the solubilization and stabilization of drugs in aqueous formulations, both for clinical use and in animal studies. Cyclodextrins consisting of 6–8 α-D-glucopyranoside units form a cone-like structure known as a toroid, with both the larger and the smaller openings of the toroid exposing the primary and secondary hydroxyl groups to the aqueous environment. This arrangement renders the interior of the toroid considerably less hydrophilic than its surroundings and is therefore capable of hosting other hydrophobic molecules. The cyclodextrin tested in this study, 2-hydroxypropyl-beta-cyclodextrin (HPBCD), is a 7-membered derivative with high aqueous solubility, an ability to form stable complexes with a wide range of compounds, and an attractive safety profile *in vivo*. HPBCD is non-hygroscopic, and does not alter the surface tension of water as much as other cyclodextrins, which can lead to a detergent-like effect with respect to biological membranes.

Our toxicity analysis of a range of HPBCD concentrations (from 0.5 to 2.5%) in embryos and larvae revealed that all developmental stages were mostly unaffected by up to 1% of this cyclodextrin derivative (Fig. 1, Table 1). Dysmorphologies were not often observed – when toxicities were seen, embyonic or larval lethality was the predominant effect.

Only one previous study has examined the biological effects of a cyclodextrin on zebrafish development – in this case, methyl-beta-cyclodextrin, which is known to inhibit endocytosis, was found to prevent the normal completion of cytokinesis in early cleavage-stage embryos [29]. Although cyclodextrins in general, and HPBCD in particular, have not yet been reported as carriers in zebrafish bioassays, a significant number of toxicological investigations have been performed in other animal models and in humans [30]. Overall, HPBCD was found to be tolerated well, especially when dosed orally, and to exhibit only limited toxicity in the animal species tested (mice, rats, dogs). In contrast to the embryotoxicity reported here in developing zebrafish, no effects on embryonic or fetal development have been observed in mammals. This incongruency may be due to differences in the absolute concentration of HPBCD to which embryos are exposed – mammalian embryos may not be exposed to the same levels of this compound that are achieved through aqueous delivery in zebrafish experiments.

### Dimethyl formamide

Dimethyl formamide (DMF) is a polar solvent with a low evaporation rate, and has a wide range of industrial uses and applications in chemistry. In biomimetics and bioengineering, DMF has been used as a solvent and a polymerization agent for tissue scaffoldings [31].

**Figure 3:**
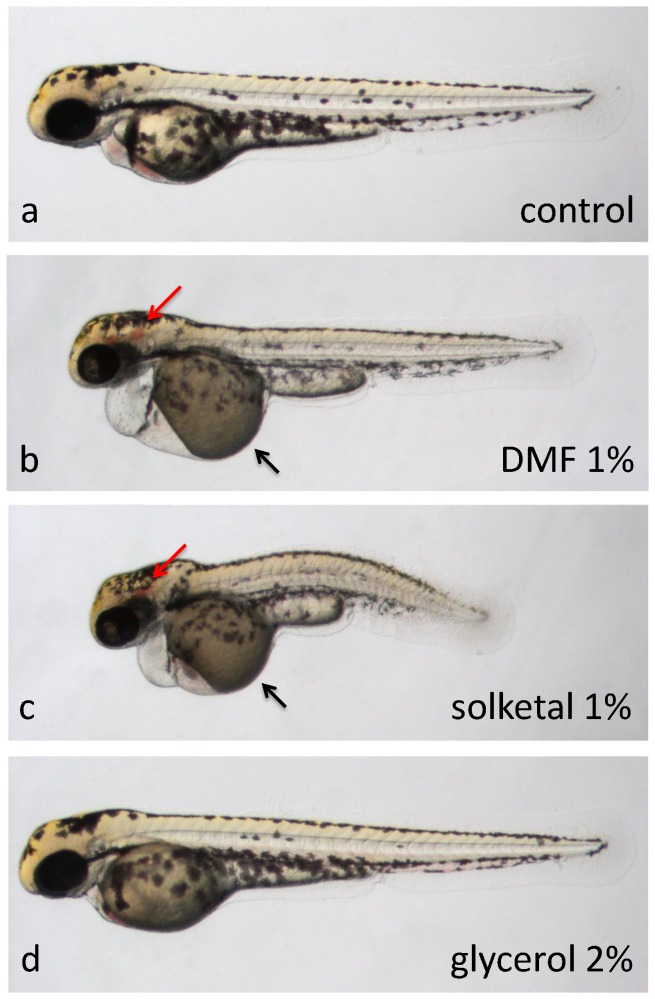
Teratogenic effects of solvents between 24 and 48 hours post-fertilization. Embryos at 2 dpf (**a, b, c, d**) treated for the previous 24 hours with **a**, embryo medium, **b**, 1% DMF, **c**, 1% solketal or **d**, 2% glycerol. Embryos in **b**, **c**, and **d** display no or very slow blood circulation as well as no touch response (all were immotile). Treatment with 1% DMF or 1% solketal also resulted in brain hemorrhaging (red arrows), pericardial edemas, yolk necrosis (black arrows) and shortening along the AP axis. **d**, Other than the observed blood flow defects, embryos treated with 2% glycerol appeared morphologically normal. All embryos are depicted with anterior to the left, dorsal to the top.

In this zebrafish study, DMF was found to be not well tolerated by either embryos or larvae, with most developmental stages revealing an MTC of either 0.5% or less (Fig. 1, Table 1). Embryos treated from 4-cell stage with 1% DMF display severe epiboly defects – eyes and some somites are still visible, but most other structures are no longer clearly discernable. Massive cell death is also present surrounding the yolk (Fig. 2b, Table 2). One-percent DMF applied from 4 hpf onwards no longer inhibits epiboly. However, embryos display body curvature and shortening, loss of ventral tail fin, no clear brain segmentation, pericardial edema and a widened choroid fissure (Fig. 2c, Table 2). Embryos treated from 1 dpf with 1% DMF display no or very slow blood circulation, no touch response, brain hemorrhaging, pericardial edemas, necrosis around the yolk and shortening along the AP axis (Fig. 3b, Table 2).

To date, no other studies have examined the embryotoxicity of DMF in zebrafish. Previous toxicological studies in rodents have reported DMF-induced liver toxicity and reduced body weight in mice and rats subjected to inhalation exposure [32]. In fish, DMF has been shown to have weak estrogenic activity, inducing vitellogenin expression in female trout [33]. Based on the results reported here, DMF is not suitable as a solvent for zebrafish experiments.

### Dimethyl sulfoxide (DMSO)

The polar solvent DMSO is the most commonly used solvent for the delivery of compounds and extracts in both cell and zebrafish-based bioassays. In cell-based assays it is normally used at concentrations not exceeding 0.1%, while zebrafish embryos and larvae are often exposed to levels of up to 1% DMSO. With its low toxicity by every route of administration and low environmental impact, DMSO has seen widespread use in pharmacological applications.

**Figure 4:**
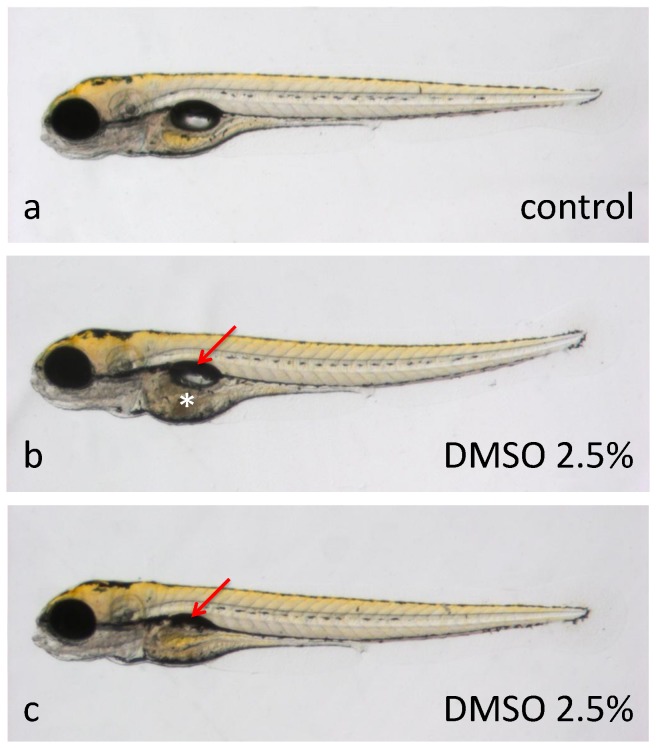
Teratogenic effects of solvents between 72 and 96 hours post-fertilization. Zebrafish embryos at 4 dpf, treated for the previous 24 hours with **a**, embryo medium, or **b** **and** **c**, 2.5% DMSO. DMSO-treated larvae display either smaller or no swim bladders (red arrows) and necrosis in the yolk area surrounding the internal organs (white asterisk). All embryos are depicted with anterior to the left, dorsal to the top.

In the zebrafish toxicity experiments performed here, DMSO was found to be tolerated well by embryos up to levels of at least 2.5% (Fig. 1, Table 1). Larvae, on the other hand, were more sensitive to higher concentrations of this solvent – in all cases beginning to reveal abnormalities or lethality in at least a subset of treated animals at DMSO levels of 2–2.5% (Table 2). Brain necrosis and swim bladder defects were observed in 3 dpf larvae treated with 2.5% DMSO (Fig. 4b,c). Loss of posture, impaired circulation and motility, and unusually bright green gall bladders were were seen in 5-dpf larvae exposed to 2.5% DMSO, and 7-dpf larvae treated with 2% DMSO displayed brain necrosis and loss of posture (Table 2).

Other groups have reported numerous studies using DMSO for the attempted cryopreservation of zebrafish embryos, and in the context of these experiments, the embryotoxicity of DMSO has been evaluated [34], [35]. Nevertheless, because of numerous differences in the composition and application of vitrification solutions to the nature of our study, these results are not directly comparable. One recent report did reveal DMSO embryotoxicity data for similar exposure protocols as used here, however the maximum DMSO concentration tested was only 2% [23]. Surprisingly, no adverse effects were observed at this concentration, in contrast to our results. One reason for these differing results may be the different strains of zebrafish that were used.

### Ethanol

While ethanol is an important solvent in chemistry and pharmacology, and is very widely used to dissolve substances intended for human contact or consumption (including not only drugs, but also scents, food colors, and flavorings), it is also a psychoactive substance and a teratogen. For these reasons, the use of ethanol as a solvent in zebrafish experiments should be considered with caution.

Our evaluation of ethanol revealed a range of MTCs from 0.5 to 1.5% over the various developmental stages analyzed (Fig. 1, Table 1). A number of phenotypes were observed, including developmental delay, cyclopia, brain segmentation and somite defects for embryos treated with 1.5% ethanol within the first 24 hours. Impaired circulation, edema, hyperactivity, and loss of posture were some of the defects seen in larvae treated with ethanol levels above the MTC (Table 2).

Ethanol has been well characterized as a teratogen in multiple species. In zebrafish, ethanol was previously found to induce several defects depending upon the developmental stage at which embryos or larvae are exposed. Zebrafish embryos, treated briefly with ethanol at early gastrula stages, developed cyclopia due in part to defects in the migration of the prechordal plate [36]. Exposure to ethanol earlier in development led to incomplete epiboly [37], whereas the acute treatment of larvae led to a dose-dependent locomotor response, with intermediate doses resulting in hyperactivity, and high doses causing hypoactivity and sedation [38]. For these reasons, the use of ethanol as a solvent in behavioral assays is not recommended.

### Glycerol

Glycerol (also referred to as glycerin) is hygroscopic and, because of three hydroxyl groups, also highly water-soluble. Because of these properties and its low toxicity, it is widely used in pharmaceutical formulations and as a humectant in food and cosmetics. In addition to its use in the cold storage of laboratory bacteria, enzymes and other biochemical reagents, glycerol is a versatile solvent – acting as a preserving agent for botanical extracts, for example, and also preventing the precipitation of tannins in ethanolic plant extracts.

We found glycerol to be well tolerated by zebrafish larvae – with 5 and 7-dpf larvae remaining unaffected at the highest concentration tested (2.5%). Younger larvae and embryos tolerated glycerol levels of either 1.5 or 2% without visible abnormalities (Fig. 1, Table 1). In larvae, higher concentrations of glycerol mainly caused impaired circulation and motility (Fig. 3d), whereas cleavage-stage embryos showed shortening of the A-P axis and *cardia bifida*, as well as somite or brain segmentation defects (Fig. 2f, Table 2).

The embryotoxicity of glycerol in zebrafish has previously been observed in the evaluation of various vitrification solutions, some of them containing glycerol at concentrations of 5% and above [35]. At the compositions tested (involving brief exposures), all cryoprotectant mixes were toxic to zebrafish embryos, including the one based on glycerol.

### Isopropanol

A widely used solvent, isopropanol dissolves a wide range of nonpolar compounds, and is also used as a disinfectant and as antifreeze. To date, the use of isopropanol as a solvent in zebrafish assays has not been reported.

In our toxicity analysis in zebrafish, we found isopropanol to have variable effects depending upon the develomental stage (Fig. 1, Table 1). In general, however, isopropanol was even less well tolerated than ethanol. Several developmental stages were adversely affected at levels above 0.5%, for example, with the most common phenotypes including impaired circulation and motility, loss of posture, and pericardial edema (Table 2).

Isopropanol, a secondary alcohol, is metabolized by alcohol dehydrogenase in the liver to generate acetone, which is not metabolized further. Like ethanol, isopropanol itself has depressive effects on the CNS, as does its primary metabolite, acetone (see above). While these effects remain to be characterized in zebrafish, it would be prudent not to use isopropanol as a solvent in behavioral assays using zebrafish larvae unless it can be clearly demonstrated that on its own, it has no discernable effect at the concentrations used.

### Methanol

A polar solvent, methanol is widely used for HPLC due to its low UV cutoff. It is also a solvent of choice for the extraction of secondary metabolites from plants and other biomaterials; crude methanolic extracts are therefore a common source of natural products for screening in bioassays.

Surprisingly, we found zebrafish larvae and embryos to tolerate up to 1.5 or 2% methanol, and in some cases even up to 2.5%, without any apparent adverse effects (Fig. 1, Table 1). Impaired circulation with seen in embryos treated with 2% methanol within the first 24 hours of development, whereas loss of posture and brain necrosis was seen in 7-dpf larvae exposed to 2.5% methanol (Table 2).

In zebrafish, methanol has primarily been investigated with respect to its potential as a cryoprotectant [34], [35]. Toxicities associated with methanol have therefore been revealed primarily in the context of this application – namely, involving the brief exposure of eggs and/or embryos to high concentrations of methanol. Beyond such studies, methanol has been found to exert its known neurodegenerative effects at least in part through the inhibition of ecto-nucleotidases and acetylcholinesterase in the zebrafish brain [39].

### Polyethylene glycol

Numerous polyethylene glycols (PEGs) of varying molecular weights are used as solvents, dispersants, and excipients for the delivery of a broad range of drugs. PEGylation, the covalent modification of pharmacologically active compounds and proteins with PEG, is a widely-used strategy to improve solubility, reduce toxicity, and improve the plasma half-life of these substances. To date, the use of PEGs in zebrafish assays has not been reported.

Our study focused on PEG-400, which was tolerated well by both embryos and larvae. All developmental stages exhibited no adverse effects up to at least 2.5% (Fig. 1, Table 1).

Although embryotoxicity studies of PEG-400 and other polyethylene glycols have previously not been reported for zebrafish, mammalian studies have been carried out. For example, rats injected intraperitoneally (i.p.) for a total of three times during the gestation period with polyethylene glycol (PEG-400) at doses equivalent to 50% of the LD_50_ (up to 30 g/kg body weight) exhibited no increase in pre-implantation mortality. However, mild signs of embryotoxicity and generally retarded development were observed [40].

### Propylene glycol

Propylene glycol is widely used as as a solvent in pharmaceutical formulations and cosmetic products, and as a food additive. The anticonvulsant diazepam, for example, which is relatively insoluble in water, contains propylene glycol in its clinical, injectable form. Propylene glycol also is one of several solvents investigated in zebrafish embryos for its ability to act as an internal cryoprotectant [34].

Like PEG-400, we found propylene glycol to be well tolerated by most developmental stages up to at least 2.5%, which was the highest concentration tested (Fig. 1, Table 1). Larvae at 3, 4 and 7 dpf showed lower MTCs of 2 or 1.5%.

Previous toxicity studies have focused on the toxicity of propylene glycol delivered to early embryos, revealing that brief exposures to cryoprotectant solutions containing between 5 and 10% of this solvent were toxic [35].

### Solketal

Solketal is a glycerol derivative, with an isopropylidene group binding two neighboring glycerols. Although not as widely used as PEG and other solvents, solketal may offer certain advantages in the preparation of pharmaceutical formulations to dissolve water- insoluble drugs [41].

Our toxicity analysis of solketal showed that embryos and larvae at most developmental stages tested did not tolerate this solvent well. Both larvae and cleavage-stage embryos showed high levels of adverse effects at the lowest concentration (0.5%), and intermediate stages showed abnormalities in the majority of embyros at solketal levels of 1% and above (Fig. 1, Table 1). Defects included developmental delay, impaired circulation and motility in embryos, and loss of posture and brain necrosis in larvae (Fig. 3c, Table 2).

Based on these results, solketal does not appear attractive as a solvent for zebrafish bioassays.

## Conclusions

While limited in scope, this toxicity screen has revealed several solvents – in addition to DMSO – that appear to have potential utility for zebrafish bioassays. Based on these initial data, polyethylene glycol (PEG-400), propylene glycol, and methanol in particular appear to be attractive solvents to deliver compounds to both embryos and larvae. In addition, acetone appears suitable for embryos. In terms of carriers, albumin (BSA) appears useful for older larvae, and cyclodextrin (HPBCD) for both embryos and larvae, albeit only up to 1% final concentration.

Several caveats will apply in the direct extension of these results to other laboratories. Appreciable differences have been observed between zebrafish strains with respect to the embryotoxic effects of ethanol [42], for example. The MTCs observed in this study, which relied on ABs, should therefore not be extrapolated to other strains such as Tübingen, TL and WIK without further experimental confirmation. Furthermore, uncharacterized environmental factors such as the water quality, diet, and stress levels experienced by adult zebrafish in different aquaculture facilities may directly or indirectly influence the sensitivity of their offspring to various chemicals such as those evalulated here. Finally, differences in the purity of solvents and carriers could influence results, depending on the grade that is used (p.a. grade solvents were used for this study). For these reasons, an initial dose-finding experiment should ideally be performed for each new solvent and carrier used by laboratories for exposing zebrafish embryos and larvae to compounds.

This study was designed to identify solvent and carriers – in addition to DMSO, which is already widely used – that might be useful in delivering small molecules to zebrafish embryos and larvae. While we were able to find apparently safe concentrations for several solvents and carriers, it is likely that these concentrations are, on their own, bioactive and therefore potentially influencing pharmacological or toxicological screens being carried out using these solvents or carriers. In general, given the relatively small differences (2–3 fold) between concentrations that are apparently safe and those that are clearly toxic, further studies – e.g. omics analyses – should be carried out to determine which cellular processes and signalling pathways are affected by any solvents and carriers that are used for small-molecule screens in zebrafish. It is interesting to note that increased hsp70 levels can be observed in zebrafish embryos and larvae exposed to sub-toxic levels of DMSO, indicating that these concentrations are capable of inducing stress reponses [43]. More recently, microarray analysis of zebrafish embryos exposed for 48 hours to low levels of DMSO revealed the differential expression of large numbers of transcripts [44], and another study detected effects on locomotor activity and behavior in DMSO-treated larvae [45]. Future studies should therefore be carried out to examine the global changes in the transcriptome and phosphoproteome of embryos and larvae treated with apparently safe concentrations of DMSO, as well as with other solvents and carriers evaluated in this study, to better understand the potential of this level of exposure to influence the bioactivity of compounds being tested. In any case, bioactivity results for individual compounds in zebrafish-based pharmacological or toxicological screens should therefore be interpreted together with the caveat that the solvent or carrier used to deliver these compounds may have influenced these results based on their own bioactivity, and that negative controls (with this solvent or carrier only) may not be sufficient to control for these effects. One solution to this problem would be to confirm the bioactivity of compounds using additional solvents or carriers for their delivery.
